# Anatomical basis of retrograde thoracic veins flow and its implications in complex thoracic wall reconstructive surgery

**DOI:** 10.1007/s00276-022-03015-5

**Published:** 2022-09-21

**Authors:** Barbara Buffoli, Vincenzo Verzeletti, Vittoria Gabusi, Lorena Giugno, Lena Hirtler, Gianpaolo Faini

**Affiliations:** 1 Anatomy and Physiopathology Division, Department of Clinical and Experimental Sciences, University of Brescia, V.le Europa 11, 25123 Brescia, Italy; 2 Interdipartimental University Center of Research “Adaptation and Regeneration of Tissues and Organs (ARTO)”, University of Brescia, 25123 Brescia, Italy; 3 Department of Cardiac, Thoracic, Vascular Sciences and Public Health, University of Padova, Padua, Italy; 4 Division of Anatomy, Center for Anatomy and Cell Biology, Medical University of Vienna, Vienna, Austria; 5 Unit of Plastic and Reconstructive Surgery, “Ospedale di Esine”, Esine, Italy

**Keywords:** Internal thoracic veins anatomy, Retrograde flow, Valves, Thoracic wall reconstruction, Free flap reconstruction

## Abstract

**Purpose:**

Internal thoracic veins are increasingly used as recipient’s vessels in chest wall reconstructive surgery due to their predictable anatomy and to the possibility to make a double venous anastomosis, exploiting the retrograde flow within them. Over the years, retrograde flow had been explained by the absence of valves in internal thoracic veins, which have been found recently instead. Therefore, our aim is to analyze the retrograde flow and its relationship with valves in the internal thoracic veins.

**Methods:**

We evaluated 32 internal thoracic veins of 16 fresh-frozen specimens with undamaged thoracic cages by dynamic analysis focused on retrograde flow assessment through a partial external circulation system obtained cannulating the subclavian veins. Gross anatomical and morphological evaluations about the presence of valves and their pattern were then made.

**Results:**

Efficient, partial, and absent retrograde flow was, respectively, found in 17/30, 8/30 and ITVs and 5/30 internal thoracic veins. Following Arnez’s classification, 20/32 Type I and 12/32 Type II internal thoracic veins were identified. Valves were observed in 10/16 specimens (62.50%) corresponding to 36.67% of examined veins (11/30). Three valves were found between the 2nd intercostal space and 12 valves in the 3rd intercostal space. 13/15 valves were bicuspid, 2/15 tricuspid. A significant correlation (*p* < 0.001) between the retrograde flow and the presence of valves in internal thoracic veins was observed.

**Conclusion:**

Our study suggests a possible influence of the presence and the number of valves in the efficient retrograde flow of the internal thoracic veins, suggesting that, especially for more complex cases, a preoperative or intraoperative evaluation of the chest wall drainage should be recommended.

## Introduction

The internal thoracic veins (ITVs) are the main vessels responsible for the physiological venous drainage of the anterior thoracic wall. Both right and left ITVs originate from the confluence of the inferior musculophrenic and epigastric veins, run laterally to the sternum between the parietal pleura and the deep layer of intercostal muscles, and flow into the respective brachiocephalic vein [18].

The use of these vessels for free tissue transfers in reconstructive surgery of the thoracic wall is now well established, because, compared to other potentially suitable vessels (i.e., the thoracodorsal vein), ITVs are less affected by atherosclerosis, their anatomy is predictable, and iatrogenic injury or scarring of these vessels through previous surgery and radiotherapy are rare [3, 8, 12, 14]. In addition, their position close to the lateral border of the sternum allows easier access for surgeons than other vessels and the possibility of positioning the free flap’s most vascularized part in the medial thoracic region. Typically, ITVs’ micro-anastomosis with the flap’s pedicle vein is made in the most proximal part of the ITVs exploiting the venous anterograde flow, which proceeds from caudal toward cranial to the caval system. Thus, the ITVs’ proximal tract is considered safe and reliable for anastomosis, maintaining their normal flow direction [3, 8, 12, 14].

However, the need to guarantee patients the best possible result in terms of both aesthetics and function has inevitably led to an increase of the complexity of surgical procedures, and hence, the idea arose of a double anastomosis with ITVs distal ends to gain an additional venous drainage [5, 7, 10, 17, 20]. Literature data from 1995 to 2020 support this proposal, based on the principle that there exist no venous valves in the ITVs, and therefore, retrograde flow (RF) through them is unhampered [2–4]. Especially regarding these last two points, there has never been absolute clarity for many years; in fact, no one has yet formulated any hypotheses about the mechanisms underlying the ITV’s RF and some groups even published data showing the presence of valves within ITVs [6, 9].

**Fig. 1:**
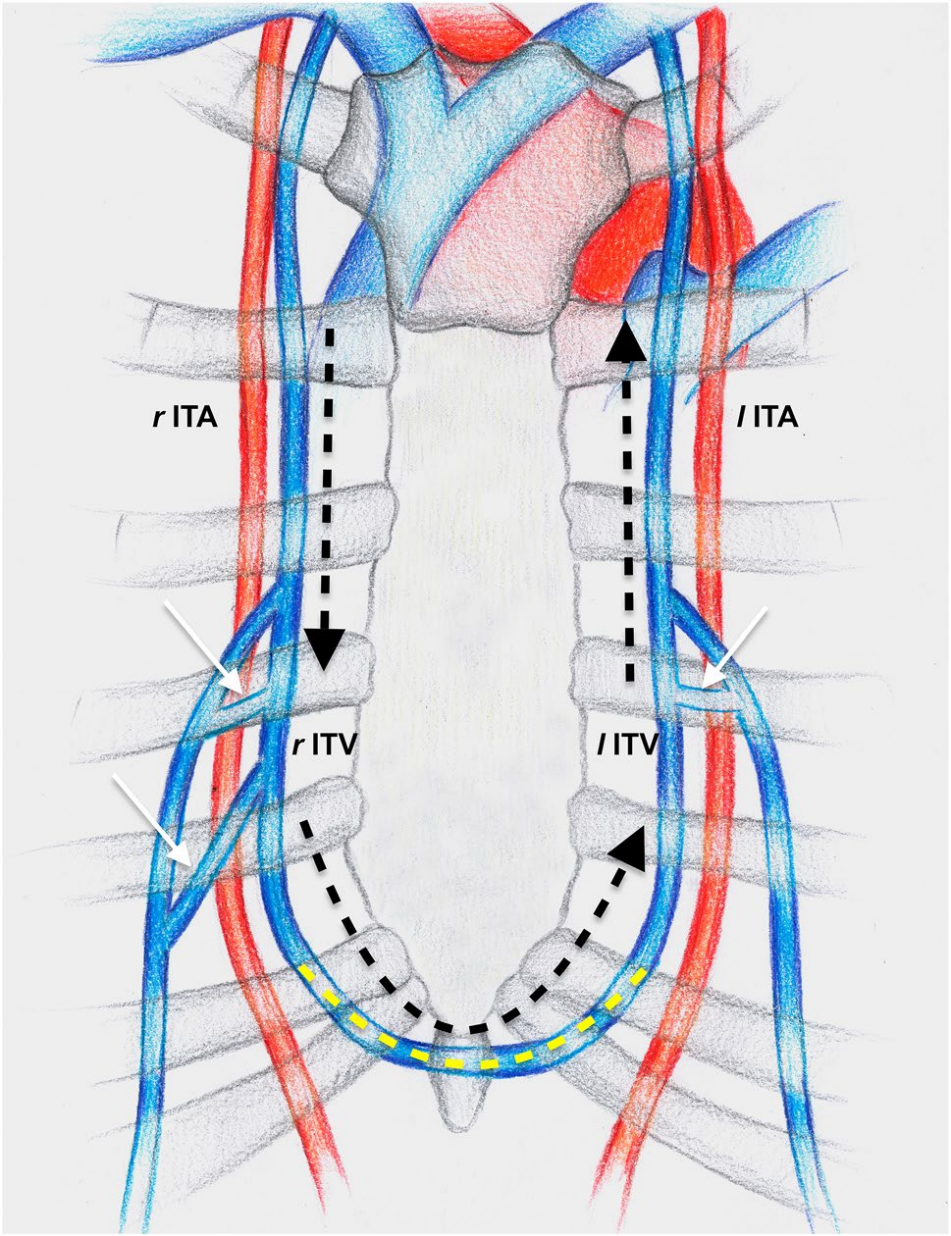
Drawing representing RF (black dotted arrows) through the right ITV to the contralateral venous system. This is allowed by a crossing vein (yellow dotted line) constantly present between the two ITVs. Black dotted arrows: RF through *r*ITV; white arrows: communicating veins below the ITVs bifurcation; yellow dotted line: crossing vein between *r*ITV and *l*ITV. *r*ITA: right internal thoracic artery; *r*ITVs: right internal thoracic vein; *l*ITVs: left internal thoracic vein; *l*ITA: left internal thoracic vein

In 2020, *Seok Nam* and colleagues published a critical study about this topic concluding on the frequent presence of valves within the ITVs (up to 55% in their series), which nonetheless had no negative impact on the ITV’s RF [15]. In addition, one of the significant contributions made by this work is the first explanation of the mechanisms underlying the RF through the ITVs, which is made possible by the presence of venous communication between the left and right ITVs; this allows the blood to flow to the contralateral side of the lower venous anastomosis and then to follow the physiological anterograde flow toward the superior vena cava [15] (Fig. 1).

All the concepts mentioned above represent the fundamentals of our study, in which the main objective is to analyze the RF within the ITV in anatomical specimens and subsequently evaluate its relationship with the presence and location of venous valves.

## Materials and methods

### Anatomical specimens

In this study, 16 thoracic walls from 16 fresh-frozen specimens (FFSs) (Caucasians, 6 males—10 females, mean age at death 82.12 years, ranging from 65 to 97 years) were examined to study the bilateral ITVs anatomy (32 ITVs, Table 1). Medical history revealed no prior surgical intervention to the thoracic wall and cavity. The specimens originated from the voluntary body donations program of the Center for Anatomy and Cell Biology of the Medical University of Vienna. The donor provided informed written consent before death for the body to use in medical education and research. The study was approved by the ethics committee of the Medical University of Vienna approved the study (Number: 1017/2016).

**Table 1:** Set of all the collected data. (i.s.—intercostal space)

Specimen	Age	Gender	Side	Patter ITV	Level of tributary vein joining	Retrograde flow	N° swelling (valves)	Swelling (valves) level	Macroscopic identification	Valve type
1	97	M	L	Type 1	Lower border 4th rib (4th i.s.)	Partial	–	–	–	–
			R	Type 2	–	Absent	Double	3rd i.s.	Positive	Bicuspid-Bicuspid
2	87	F	L	Type 1	Lower border 3rd rib (3rd i.s.)	Absent	Double	2nd i.s.	Positive	Tricuspid-Bicuspid
			R	Type 1	Lower border 3rd rib (3rd i.s.)	Efficient	–	–	–	–
3	96	F	L	Type 2	–	Not evaluable (thrombus)	–	–	–	–
			R	Type 1	Lower border 3rd rib (3rd i.s.)	Absent	Single	3rd i.s.	Positive	Tricuspid
4	84	F	L	Type 1	Upper border 5th rib (4th i.s.)	Absent	Triple	3rd rib–3rd i.s.–4th i.s.	Positive	Bicuspid–Bicuspid–Bicuspid
			R	Type 1	Lower border 3rd rib (3rd i.s.)	Efficient	–	–	–	–
5	92	F	L	Type 2	–	Not evaluable (subclavian artery aneurism)	–	–	–	–
			R	Type 2	–	Efficient	–	–	–	–
6	83	F	L	Type 1	Upper border 5th rib (4th i.s.)	Efficient	–	–	–	–
			R	Type 2	–	Efficient	–	–	–	–
7	71	F	L	Type 2	–	Partial	Single	3rd i.s.	Dubious	Bicuspid
			R	Type 2	–	Partial	Single	2nd i.s.	Dubious	Bicuspid
8	65	M	L	Type 1	Upper border 4th rib (3th i.s.)	Absent	Single	3rd i.s.	Positive	Bicuspid
			R	Type 2	–	Partial	–	–	–	–
9	86	F	L	Type 1	Lower border 3rd rib (3rd i.s.)	Efficient	–	–	–	–
			R	Type 1	Upper border 4th rib (3rd i.s.)	Partial	Single	3rd i.s.	Dubious	Bicuspid
10	91	M	L	Type 1	Lower border 4th rib (4th i.s.)	Efficient	–	–	–	–
			R	Type 2	–	Efficient	–	–	–	–
11	80	F	L	Type 1	Upper border 4th rib (3rd i.s.)	Efficient	–	–	–	–
			R	Type 1	Lower border 3rd rib (3rd i.s.)	Efficient	–	–	–	–
12	74	F	L	Type 1	Lower border 3rd rib (3rd i.s.)	Efficient	–	–	–	–
			R	Type 2	–	Partial	Single	3rd i.s.	Dubious	Bicuspid
13	86	M	L	Type 1	Lower border 3rd rib (3rd i.s.)	Efficient	–	–	–	–
			R	Type 2	–	Partial	Single	3rd i.s.	Dubious	Bicuspid
14	78	M	L	Type 1	Lower border 3rd rib (3rd i.s.)	Efficient	–	–	–	–
			R	Type 2	–	Efficient	–	–	–	–
15	72	M	L	Type 1	Upper border 4th rib (3rd i.s.)	Partial	Single	3rd i.s.	Dubious	Bicuspid
			R	Type 1	Lower border 4th rib (4th i.s.)	Efficient	–	–	–	–
16	72	F	L	Type 1	Upper border 4th rib (3rd i.s.)	Efficient	–	–	–	–
			R	Type 1	Upper border 4th rib (3rd i.s.)	Efficient	–	–	–	–

### External circulation system and retrograde flow evaluation

We evaluated 32 ITVs of 16 fresh-frozen specimens with undamaged thoracic cages by dynamic analysis focused on retrograde flow assessment through a partial external circulation system. Two (2/32, 6.25%) subclavian veins were not appropriate for the application of the system: in the first case due to the presence of a huge subclavian artery aneurism, compressing the subclavian vein and obstructing the flow and, in the second case, due to the presence of a full-thickness thrombus.

**Fig. 2:**
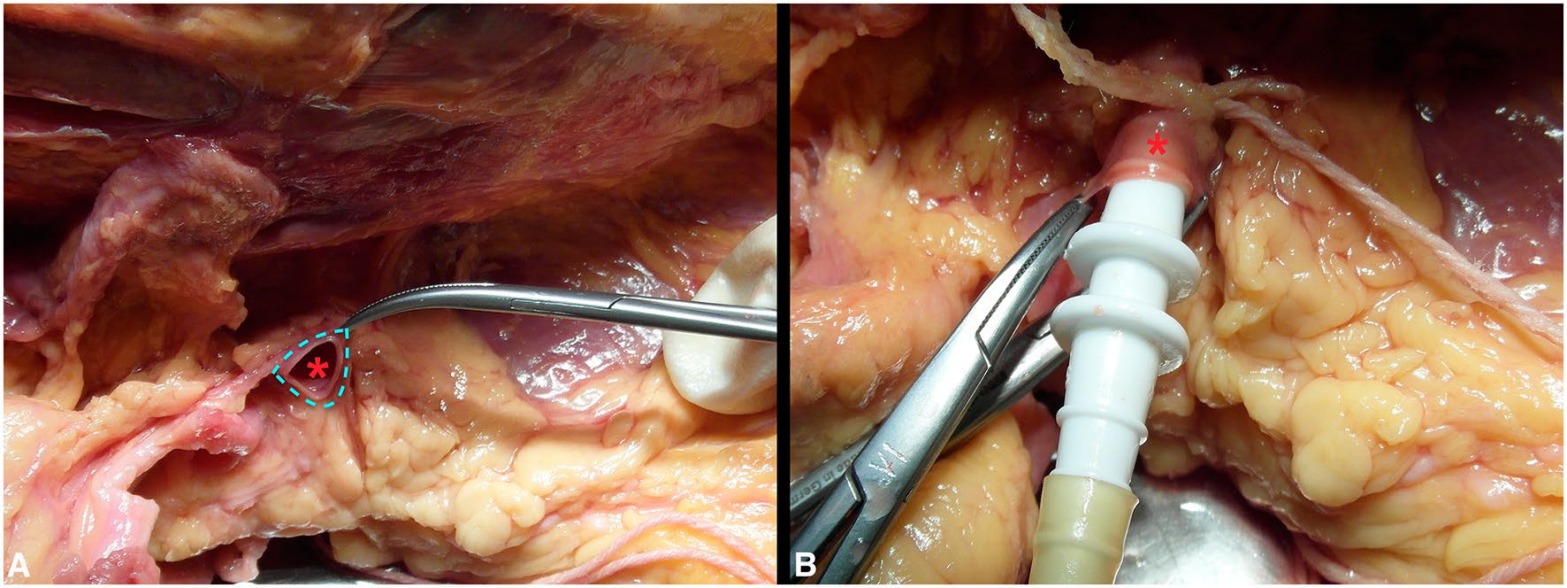
Dynamic simulation of RF within the FFSs venous system. **A** Identification of the subclavian vein (light blue dotted line) for each side of each FFSs. **B** Cannulation of the subclavian vein and start with the venous perfusion. Red asterisk (*): left subclavian vein

The dynamic external circulation system was set up by the intravenous cannulation of the subclavian veins of the specimens, which were subsequently connected to an arthroscopy pump (Arthrex, München, DE), able to maintain a stable infusion pressure of 30 mm/Hg of a saline solution (9% NaCl). The cannulation of the right subclavian vein ensured flow into the right ITV; the same concept applies to the opposite side (Fig. 2). Before starting the infusion, both ITVs of each specimen were isolated through an external parasternal approach, isolating the vessels from the parietal pleura and intercostal muscles, so that the vessel could be observed in its integrity. Afterward, the ITVs were cut at the level of the upper border of the 6th rib.

The RF was analyzed in 2 steps:

1. Dynamic evaluation: in all the specimens, we evaluated the filling degree of the ITVs and the outflow of the solution from the caudal part of the sectioned ITV.

**Fig. 3:**
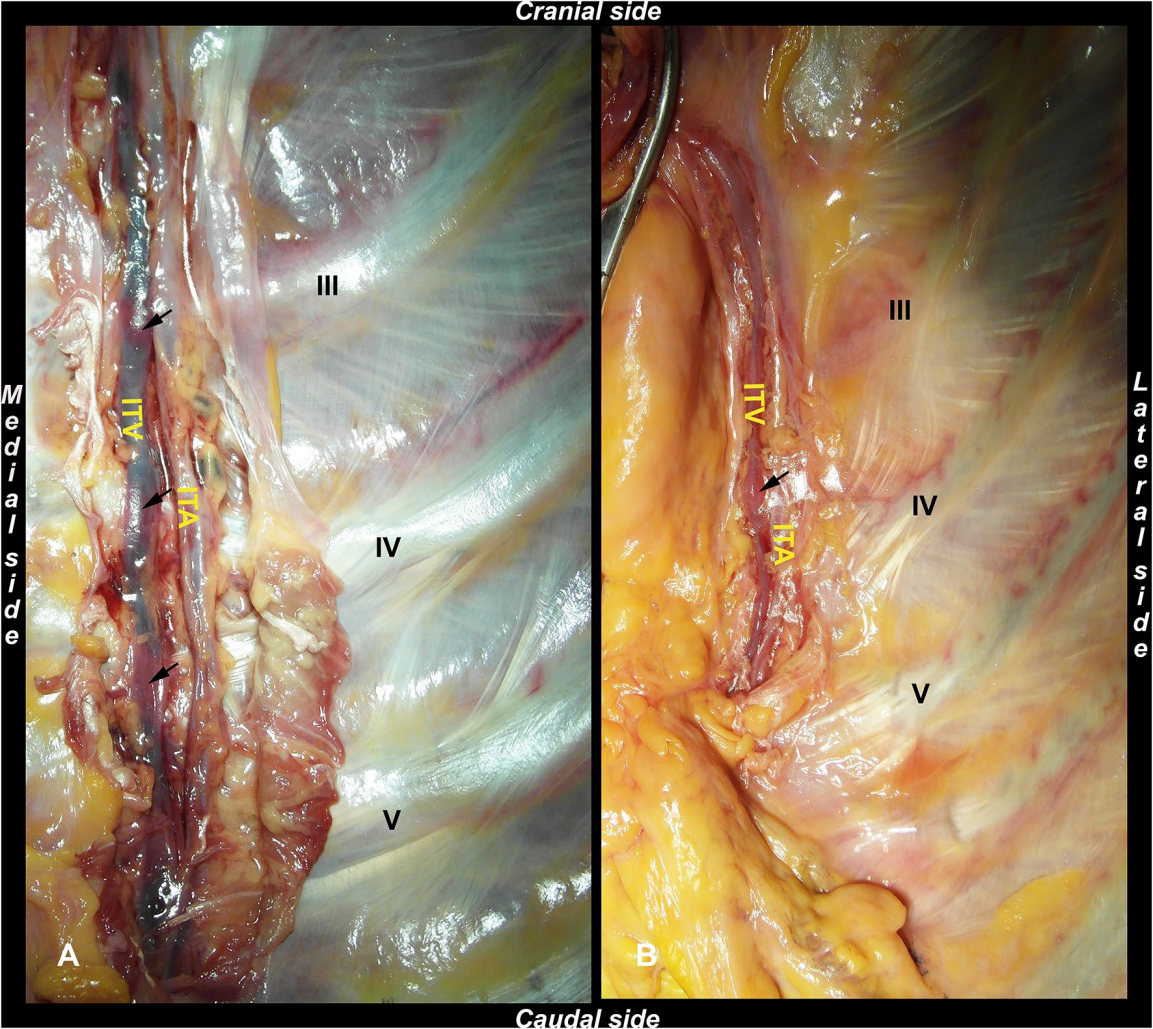
Swellings identification through RF static evaluations. **A** Triple swelling (black arrows) in a Type II ITV. **B** Single swelling (black arrow) observed in a Type I ITV. ITV: internal thoracic vein; ITA: internal thoracic artery. III, IV, V: 3rd, 4th, and 5th rib

The veins flow and filling were judged as:

- Efficient: complete filling of the vein and complete flow.
- Partial: partial filling of the vessel and reduced flow.
- Absent: no filling of the vessel and absence of flow.

2) Static evaluation: in those ITVs where both flow and filling were judged to be partial or reduced, we identified the tracts of veins presenting possible congestion, observing single or multiple swellings (Fig. 3).

The results of the two analyses were documented for both sides of each specimen.

### Assessment of ITVs’ pattern and samples collection

**Fig. 4:**
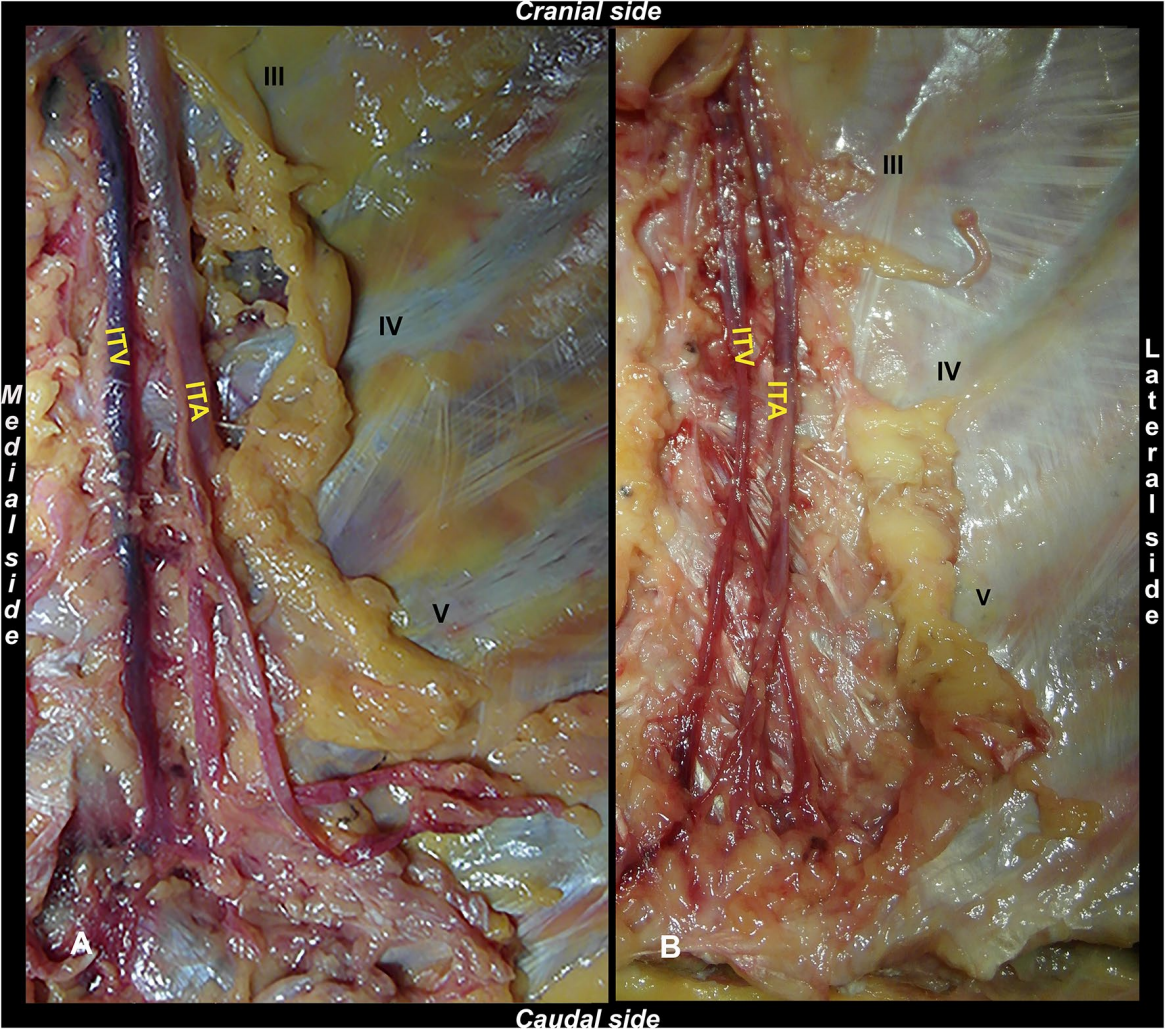
ITV types according to Arnez et al*.*’s classification. **A** Type II ITV. **B** Type I ITV, with bifurcation (black arrow) at the level of the 4th intercostal space. ITV: internal thoracic vein; ITA: internal thoracic artery. III, IV, V: 3rd, 4th, and 5th rib

To facilitate anatomical analysis, the anterior chest wall was removed *en-bloc* after completion of the retrograde flow evaluation. Thus, it was possible to complete the isolation of the structures of interest, which was already started during the RF assessment. The internal thoracic artery (ITA) and the ITV were dissected by a gentle and blunt dissection, and the main collateral branches and tributaries were ligated. The gross anatomical evaluation was focused on identifying the different patterns of ITVs, according to the classification of Arnez and colleagues [2] (Fig. 4):

- Type I: ITV is medial to the ITA and was subdivided into two interconnecting veins at the level of the 3rd or 4th intercostal space,
- Type II: ITV is medial to the ITA without bifurcation,
- Type III: ITV is lateral to the ITA with a subdivision into two interconnecting veins at the level of the 3rd or 4th intercostal space,
- Type IV: ITV is lateral to the ITA without bifurcation.

Images and notes of the arterial and venous patterns were collected, with respect to the ribs and the intercostal spaces. Afterward, the ITVs were sectioned at the level of the 1st and 5th intercostal spaces and harvested for further analysis.

### Morphological evaluation

**Fig. 5:**
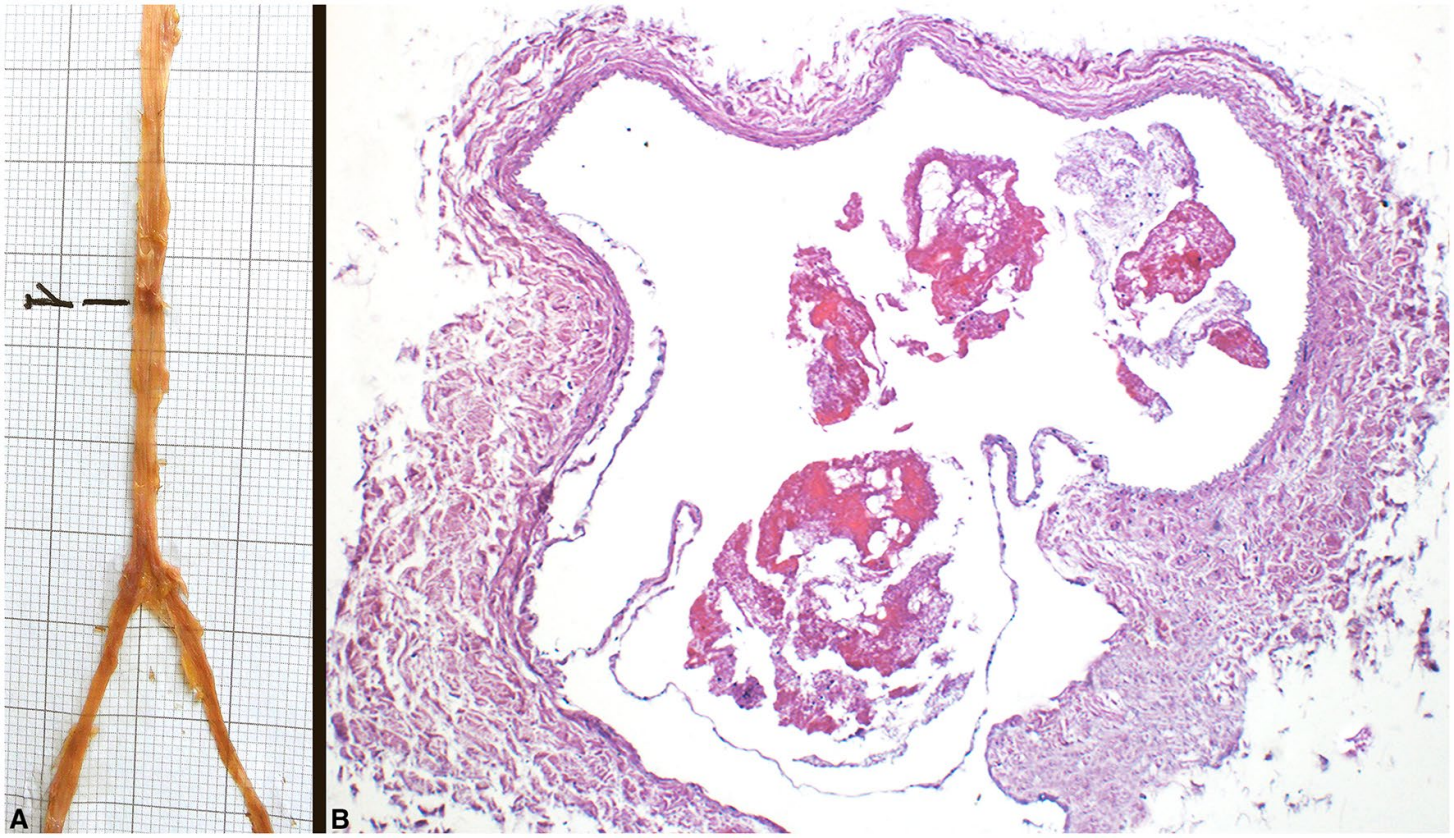
Morphological evaluation of the ITVs valves. **A** ITV macroscopic evaluation and identification of a single valve (1). **B** Microscopic evaluation (10x) of a valve

The harvested ITVs were processed for morphological evaluations involving macroscopic and microscopic investigations. The macroscopic analysis looked for the presence of any valves, identifiable to the naked eye, whose location was mapped. For the microscopic investigations, all samples were divided into shorter sections containing the level of the previously observed swelling, fixed in a 10% neutral buffered formalin solution, and embedded in paraffin using automatic tissue processor (Donatello Series 2, Diapath BG, Italy); 5 µm-thick sections were cut by microtome (Semi-automatic Rotary Microtome Galileo, Diapath, BG, Italy) and collected on poly-L-lysine coated glass slides; the sections were cut transversally to the longitudinal axis of the vessel. Sections were deparaffinized in xylene, rehydrated in descending alcohol concentrations, and stained with Haematoxylin–Eosin and Masson–Goldner staining using automatic stainer (Giotto, Diapath, BG, Italy). The microscopic slides were then observed under a light microscope at 4×, 10× and 20× magnification (Olympus, Milan, IT), connected to a computer equipped with image analysis software (Image-Pro Premier 9.1, Immagini e Computer, Milan, Italy) (Fig. 5). Both macroscopic and microscopic evaluations were performed by two operators unaware of the processed samples. Thus, the presence of valves that were not visible to the naked eye (dubious) was microscopically assessed and they were subsequently mapped according to their anatomical position.

### Statistical analysis

Statistical analysis was performed to assess the existence of a correlation between:

1. RF and number of valves (Spearman’s correlation test);
2. RF and ITVs’ pattern (Fisher’s exact test);
3. presence of valves and ITVs’ pattern (Fisher’s exact test);
4. RF and FFSs’ gender (Fisher’s exact test);
5. And RF and FFSs’ age (Spearman’s correlation test).

A *p* value of below 0.05 was deemed statistically significant.

## Results

### External circulation system and retrograde flow evaluation

The evaluation of the RF from the ITVs after continuous infusion through the ipsilateral subclavian vein showed 17 ITVs (17/30, 56.67%) where the outflow was judged complete and therefore with efficient RF, whereas 8 ITVs (8/30, 26.67%) presented partial RF and 5 ITVs (5/30, 16.67%) did not show any outflow and the RF was therefore judged as absent. During the static evaluation, 11 ITVs (11/30, 36.67%) in 10 FFSs (10/16, 62.50%) presented at least one swelling: 8 single swelling (8/11, 72.7%), 2 double swellings (2/11, 18.18%), and 1 triple swelling (1/11, 9%) were observed, for a total amount of 15 swellings. All these data are summarized in Table 1 and Table 2.

**Table 2:** Retrograde blood flow analysis

	Retrograde blood flow (*N* = 30)				
	Type 1 (*N* = 20)		Type 2 (*N* = 10)		
Side	Left (%)	Right (%)	Left (%)	Right (%)	Total (%)
Efficient	8 (40)	5 (25)	0 (0)	4 (40)	56.67
Partial	2 (10)	1 (5)	1 (10)	4 (40)	26.67
Absent	3 (15)	1 (5)	0 (0)	1 (10)	16.67

### Assessment of ITVs’ pattern

All 32 ITVs were evaluated, and two different patterns were identified according to Arnez et al*.* [2]. In our series, the most common one was Type I ITVs (20/32, 62.5%), whereas Type II ITVs was noted in 12 ITVs (12/32, 37.5% of the cases).

Thereafter, we identified the point of the Type I ITV’s bifurcation in relation with the intercostal spaces. The veins with bifurcation (*N* = 20) never divided over the superior border of the 3rd rib. Fifteen ITVs (15/20, 75%) divided into the 3rd intercostal space at the level of the inferior border of the 3rd rib (9/20, 45%) and the superior border of the 4th rib (6/20, 30%). The other 5 ITVs (5/20, 25%) divided into the 4th intercostal space at the level of the inferior border of the 4th rib (3/20, 15%) and the superior border of the 5th rib (2/20, 10%).

All the data are reported in Table 1.

### Morphological evaluation

All observed swellings (15/15, 100%) revealed the presence of the venous valves. Valves were observed in 10/16 (10/16, 62.50%) thoracic walls. Nine valves (9/15, 60%) were positive at the macroscopical identification, whereas six valves (6/15, 40%) were dubious and were confirmed later after microscopic evaluation.

Among the ITVs, 11 (11/30, 36.66%) presented at least one swelling (valve), with a higher predominance in females (7/10, 70%) and a lower distribution in males (4/6, 66.66%). Three valves (3/15, 20%) were in the 2nd intercostal space; 12 valves (12/15, 80%) were in the 3rd intercostal space. Microscopic analysis revealed the presence of 13 bicuspid valves (13/15, 86.66%) and 2 tricuspid valves (2/15, 13.33%).

All the above-mentioned data are reported in Table 1.

### Statistical analysis

#### Correlation between retrograde flow and the presence of valves

There was a statistically significant association between the type of RF and the presence of valves as assessed by Spearman’s correlation test (*p* value =  < 0.001).

An efficient flow was observed in 17 ITVs; in all of these (17/17, 100%), no valves were noted. Among the eight ITVs with a partial retrograde flow, 6 ITVs (6/8, 75%) presented single valves and 2 ITVs (2/8, 25%) had no valves. We found 5 cases of absent outflow concomitant with 1 case of triple valves (1/5, 20%), 2 cases of double valves (2/5, 40%), and 2 cases of single valves (2/5, 40%).

#### Correlation between retrograde flow and ITV pattern

There was not a statistically significant association between RF and ITVs pattern as assessed by Fisher’s exact test (*p* value = 0.129).

Efficient flow was noted in 13 ITVs of Type I (13/20, 65%) and in 4 ITVs of Type II (4/10, 40%). Partial flow was present in 3 ITVs of Type I (3/20, 15%) and in 5 ITVs of Type II (5/10, 50%). Absent flow was found in 4 ITVs of Type I (4/20, 20%) and in 1 ITVs of Type II (1/10, 10%).

#### Correlation between ITVs pattern and presence of valves

There was not a statistically significant association between the presence of valves and ITV pattern as assessed by Fisher’s exact test (*p* value = 0.703).

Among the 20 ITVs of Type I, 14 (14/20, 70%) presented no valves, 4 (4/20, 20%) presented a single valve, 1 (1/20, 5%) had double valves, and 1 (1/20, 5%) had triple valves. Among the 10 ITVs of Type II, seven (5/10, 50%) did not present any valves, four (4/12, 40%) presented a single valve, and one (1/10, 10%) had double valves.

#### Correlation between retrograde flow and specimen(s) gender

There was not a statistically significant association between RF and FFSs’ gender as assessed by Fisher’s exact test (*p* value = 0.874).

Among the 17 ITVs where the RF was judged as efficient, six (6/17, 35.29%) were observed in males, whereas 11 (11/17, 64.71%) in females. Among the eight thoracic walls where the RF was noted as partial, four (4/8, 50%) were males and four (4/8, 50%) were females. Absent flow was found in two (2/5, 40%) males and in three (3/5, 60%) females.

#### Correlation between retrograde flow and specimen(s) age

There was not a statistically significant association between the type of RF and the age of the body donors as assessed by Spearman’s correlation test (*p* value = 0.864).

The average age of the body donors where efficient RF was observed was 81.70 years; the median was 83 years. The average age of body donors where partial RF was noted was 86.20 years; the median was 87 years. The average age of body donors with an absent RF was 77.75 years; the median was 73 years.

## Discussion

The main advantage of choosing ITVs for chest wall reconstruction is related to the possibility of performing a double venous micro-anastomosis, thus exploiting the RF within them. There are well-established circumstances in which the use of an accessory venous anastomosis is determinant to graft survival, considering that the predominant cause of free flap failure is venous outflow failure: (1) when multiple flaps are required, (2) to boost blood flow for supercharging purposes, and (3) as salvage recipient vessels when the antegrade flow of ITVs is not available [5, 15]. Although the venous double anastomosis technique is often proposed in surgical practice, in recent years, there has been a heated debate in the literature about whether valves are present in the ITVs and whether the presence of valves could affect ITV’s RF. In the most recent studies, mainly when morphological investigations have been carried out, it has emerged that valves are present within ITVs with an estimated frequency of between 40 and 60% [1, 9, 15]; these data have also been confirmed in our series. This concept represented the foundations of the present study, which subsequently evaluated other factors possibly influencing RF within ITVs, thus trying to characterize some of them as predictors of flow. However, it seems that the presence and the number of valves within the ITVs represent the only determining factor in this, as sex, age, and type of vein observed in the body donors of this study did not reveal any statistical significance. This, however, is also worth mentioning, as it does not seem possible to select patients who could be good candidates for this technique, by taking only these factors into account.

Thus, considering the valves as the only factor able to influence RF, our findings deviate from those found in the literature, especially regarding their position. As a matter of fact, we found a considerable percentage (50%) of valves in the 3rd intercostal space respect to the 2nd one, as previously reported in other studies [9, 15]. This aspect is not negligible, since usually the double venous anastomosis is performed in the 2nd intercostal space, and the presence of valves below this level might have the potential for flow disturbance [15]. It is essential to emphasize the conditional verb in the previous sentence, since there are several clinical studies reporting high success rates of grafts in which RF was exploited, but which did not analyze the presence or absence of valves within the ITVs. Now, it is unlikely to think that, given the frequent presence of valves confirmed by more recent studies including ours, valves were not also present in the previously mentioned series [7, 16, 17, 20].

The central hypothesis regarding the simultaneous presence of RF and valves within a single ITV may be related to a combination of 2 factors: valve insufficiency and ITV tributaries able to bypass the valves [9, 19]. In this regard, it is interesting to point out that our data show, for the first time, that the presence of more than one valve in a single ITV (i.e., double or triple valves) is strictly linked to a dramatic reduction of RF and this may also interfere with the competence of the valves themselves. In our opinion, therefore, the presence of more than one valve in the same vessel can increase the continence mechanism of these valves, thus affecting the quality of RF within them.

The main limitation of our study is the fact that we did not evaluate the tributaries between a single ITV after its bifurcation or the crossing veins between left and right ITVs [15]; this does not allow us to exclude that RF can bypass the obstacle represented by continent valves through these collaterals or crossing veins.

In the light of the above, preoperative or intraoperative venous drainage evaluations, such as the ICG fluorescent angiography, duplex Doppler sonography, and pressure or velocity measurements, should be recommended, especially for those cases where the additional double anastomosis and RF are essential for graft survival [5, 11, 13].

## Conclusion

RF within ITVs is influenced by the presence and number of venous valves. Other factors, such as age, sex, and gender, do not influence RF. 10/16 specimens (62.50%) corresponding to 11/30 veins (36.67%) had at least one valve without a significant different distribution respect to the body side; moreover, multiple valves were observed in 3/16 specimens (18.75%) equivalent to 3/30 veins (10%). These data suggest the importance of preoperative or intraoperative study of venous drainage during reconstructive surgery of the thoracic wall.
